# Does Native Capillary Zone Electrophoresis-Mass Spectrometry Maintain the Structural Topology of Protein Complexes?

**DOI:** 10.1021/acs.analchem.4c06949

**Published:** 2025-04-01

**Authors:** William J. Moeller, Zihao Qi, Qianjie Wang, Qianyi Wang, Vicki H. Wysocki, Liangliang Sun

**Affiliations:** † Department of Chemistry and Biochemistry, The Ohio State University, Columbus, Ohio 43210, United States; ‡ Native MS Guided Structural Biology Center, The Ohio State University, Columbus, Ohio 43210, United States; § Department of Chemistry, Michigan State University, 578 S Shaw Lane, East Lansing, Michigan 48824, United States

## Abstract

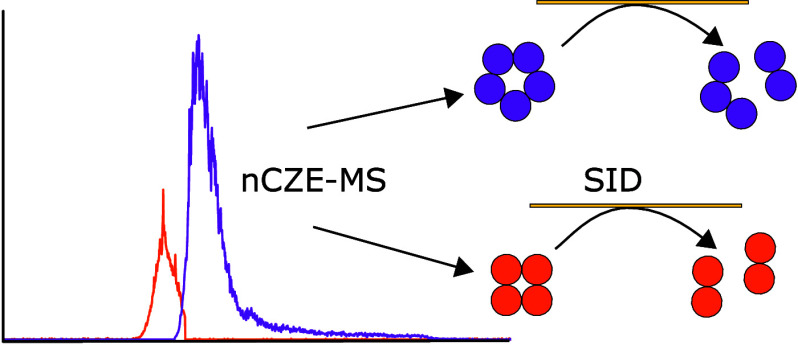

Native capillary zone electrophoresis-mass spectrometry (nCZE-MS) is a useful analytical tool for studying protein complexes. However, the extent to which the protein complexes maintain their native structural topology in nCZE-MS compared to traditional native MS (nMS) is still not fully characterized. In this technical note, we contribute to this topic by coupling nCZE-MS with surface-induced dissociation (SID) for two well-studied protein complexes (streptavidin and human recombinant C-reactive protein). SID cleaves the weakest interface of a given complex, making it a powerful diagnostic tool for identifying perturbations of protein complex structures based on fragmentation patterns. The SID fragmentation patterns of the two protein complexes from nCZE-MS under normal and charge-reducing conditions show a high similarity index compared to those from direct infusion. Additionally, nCZE-MS shows the potential to separate different conformations or different proton distributions that have subtle differences. Although only two cases are shown, the results suggest that nCZE-MS can maintain the native-like structural topology of protein complexes.

## Introduction

Capillary zone electrophoresis (CZE) has shown its potential for separating biomolecules (i.e., proteins) under physiological conditions since its introduction in the 1980s.^1^ Native CZE-mass spectrometry (nCZE-MS) was used to separate and measure several protein complexes from a red blood cell lysate in 2008.^2^ Over the last 10 years, multiple research groups have explored nCZE-MS as a powerful analytical tool for the characterization of simple mixtures of protein complexes, such as a mixture of standard protein complexes,^3−5^ nucleosomes,^6^ ribosomes,^7^ and other systems.^8−11^ Due to the technological improvements in CE-MS interfaces, capillary coating, and mass spectrometers,^12,13^ native proteomics of whole cell lysates has also been investigated.^14,15^ However, these studies did not show how well nCZE-MS maintains the structural topology of protein complexes during the measurement. This is a long-standing fundamental question in the nCZE-MS field, which has not been fully evaluated.

In this technical note, we offer a pilot study to answer this fundamental question by coupling nCZE-MS with surface-induced dissociation (SID). SID activates protein ions by colliding them with a stationary surface through a rapid, single-step collision with high energy deposition.^16,17^ Collision-induced dissociation (CID) involves colliding protein-complex ions with neutral gas molecules/atoms multiple times, typically involving restructuring of the given complex with release of a highly charged monomer. By contrast, SID fragments macromolecular complexes with charges distributed between subunits in a manner expected based on their size and structure and along their weakest interfaces.^16,18^ SID fragments correlate with interfacial strength and, ultimately, indicate the topology of large biomolecular complexes of different sizes, topologies, and compositions.^18,19^ Because SID has been shown to reflect interfacial properties, it can also be used to determine if the structure of a given protein complex has been perturbed either in solution^20^ or in the gas phase.^21−23^ Hence, SID is advantageous as a diagnostic technique for disrupted native topology. Here, we used two well-studied protein complexes, i.e., streptavidin (SA) and human recombinant C-reactive protein (CRP), as the model complexes to study structural changes during nCZE-MS analysis under various experimental conditions by SID. CRP is useful because it is known to collapse and then expand under activating conditions, leading to an increased abundance of monomers of higher charge state,^22,23^ and streptavidin is known to produce dimers from its dimer-of-dimers structure when native and trimers plus highly charged monomers when non-native.^23,24^

## Experimental Section

### Sample Preparation

Streptavidin (SA) was purchased from Thermo Scientific Pierce (Rockford, IL, USA) and human recombinant C-reactive protein (CRP) from Millipore Sigma (St. Louis, MO, USA). Both SA and CRP were buffer-exchanged into 50 mM ammonium acetate (AmAc, Millipore Sigma) by a 6-kDa cutoff gel chromatography spin column from Bio-Rad (Hercules, CA, USA). The *P*6̅ column was rinsed with 50 mM AmAc three times. The given protein complex was then pipetted into the prepared column and buffer exchanged twice. For charge reduction experiments, 20% (v/v) 50 mM triethylammonium acetate (TEAA, Millipore Sigma) was added.^25^

### nCZE-MS

The nCZE-MS system was set up by coupling a Beckman CESI 8000 Plus (Sciex) and a CE-MS interface from CMP Scientific to a Q Exactive Ultra High Mass Range (UHMR) Orbitrap Mass spectrometer (ThermoFisher Scientific) modified with a 4 cm, 12-lens SID device described elsewhere.^26^ A fused silica capillary (50/360 μm i.d./o.d., 1 m long) was coated with linear polyacrylamide (LPA) according to a previous procedure.^27^ Briefly, the bare fused capillary was pretreated by rinsing with 1 M sodium hydroxide, water, 1 M hydrochloric acid, water, and methanol, followed by incubation with 3-(trimethoxysilyl) propyl methacrylate for 3 days. After degassing with nitrogen, the capillary was filled with degassed acrylamide solution (4% in water) containing ammonium persulfate and then incubated at 50 °C for 55 min. The capillary was flushed with water to remove the unreacted reagents. One end of the LPA capillary was etched with hydrofluoric acid for 85 min to reduce the outer diameter to around 100 μm. Unless specified, for the normal charge conditions, 50 mM ammonium acetate (AmAc) was used as the background electrolyte (BGE) (pH around 6.8), while for charge-reducing conditions, 40 mM AmAc with 10 mM TEAA was used instead. 50 nL of protein mixtures (0.5 μM of each complex) were injected using 5 psi for 9.5 s and separated under 20 kV separation voltage and 1 psi pressure. The CESI 8000 and UHMR were interfaced with a commercial electro-kinetically pumped sheath flow CE-MS interface (CMP Scientific).^28^ The sheath buffer contains 10 mM AmAc, and the electrospray voltage was set to 1.9 kV, which was consistent across all experiments. To transport large biomolecules into the gas phase, the MS was tuned to the following settings, which were maintained throughout the acquisition, capillary temperature: 250 °C and −30 V in-source trapping for better declustering and deadducting; ion transmission: high *m*/*z*; detector optimization: low; trap gas flow: 5 (UHV readback shows approximately 10^–11^ mbar); mass resolution: 6,250 (400 *m*/*z*).

### Isolation and SID

The home-built SID device is not currently set up for automated data-dependent acquisition (DDA). Therefore, we performed 3 individual CZE-MS separations to collect MS1, isolation, and fragmentation data, respectively, for our proteins of interest under both normal charge and charge-reducing conditions. The first run consists of standard MS1 and is used to determine the migration times of each protein complex. We then use that information to set isolation windows around our peaks of interest that align with the expected migration times. A schematic of this separation can be found in Figure S1.

Odd charge states were selected to facilitate data interpretation because SID gives symmetric charge partitioning.^29,30^ Charge states of 15+ for SA and 23+ for CRP were isolated for the normal charge conditions. In contrast, 11+ SA and 17+ CRP were isolated for dissociation for the charge-reducing conditions. Home-built software controls all the voltages on the 4 cm SID device. For normal charge conditions, SID 45 V was applied, which corresponded to 675 eV for SA and 1035 eV for CRP. To approximately normalize the energy, SID 65 V was used for the charge-reducing conditions, which corresponded to 715 eV for SA and 1105 eV for CRP.

### Data Analysis

All mass and MS/MS spectra were processed and deconvolved by UniDec 7.0.1 with the default settings apart from the following: sample mass every 1 Da, automatic *m*/*z* peak width was used.^31^ A home-built/custom Python script was used to replot the electropherograms, raw mass spectra, and deconvolved mass spectra by UniDec for visualization purposes.

## Results and Discussion

We first optimized ionic strength and separation conditions for nCZE-MS. Native MS employs volatile salts to reduce gas phase adducting while preserving the native structure of the analyte of interest. AmAc has become a staple in nMS as it allows for minimum adduction at physiologically relevant pHs and ionic strengths,^32^ although some complexes may not retain native structure in ammonium acetate,^33^ especially at “normal charge”.^25,34,35^ As the charges of proteins have been shown to relate to their native-like structure in the gas phase,^17,36,37^ we have employed the use of a charge-reducing reagent, TEAA, that, when added to a sample, results in a lower gas phase charge of the protein. nCZE-MS necessitates the use of lower ionic strength solutions than are typically used in nMS experiments because the high separation voltages generate high currents and Joule heating when the ionic strength is high, potentially perturbing the proteins’ structure.^5^ We, therefore, compared the effect of reduced ionic strengths (20 mM and 50 mM) on the peak area via direct infusion (Figure S2). With the solution only containing AmAc, the peak area was not affected by the ionic strength. However, after TEAA was introduced at lower ionic strength (20 mM), the signal was insufficient for MS/MS experiments within the time window for CZE separation, likely due to poorer desolvation. To balance this with the possibly high current generated from high ionic strength, for the normal charge conditions, 50 mM AmAc was used as the BGE of CZE, while for the charge-reduced condition, 40 mM AmAc with 10 mM TEAA was used as the BGE to maintain similar conductivity and overall ionic strength. Additionally, the peak area decreased around 1 order of magnitude for SA after TEAA was introduced, while the intensity of CRP remained the same.

The ability of nCZE-MS to separate protein complexes from a highly complex mixture has been shown recently.^4,6,7,14,38^ Here, we mixed SA, a dimer of dimers, and CRP, a cyclic pentamer, to study maintenance of their native-like structure in nCZE-MS. The choice of these protein standards was influenced by the need for highly predictable fragmentation patterns as both are homo-oligomeric protein complexes whose ion mobility behavior and fragmentation by SID have been well characterized.^18,25,26,39,40^ SA and CRP also represent proteins with different oligomeric states and different quaternary structures, providing validation while minimizing confounding variables. Both complexes are also widely used as standards in nMS, and this work can serve as a benchmark for future experiments involving nCZE-MS.

**Figure 1:**
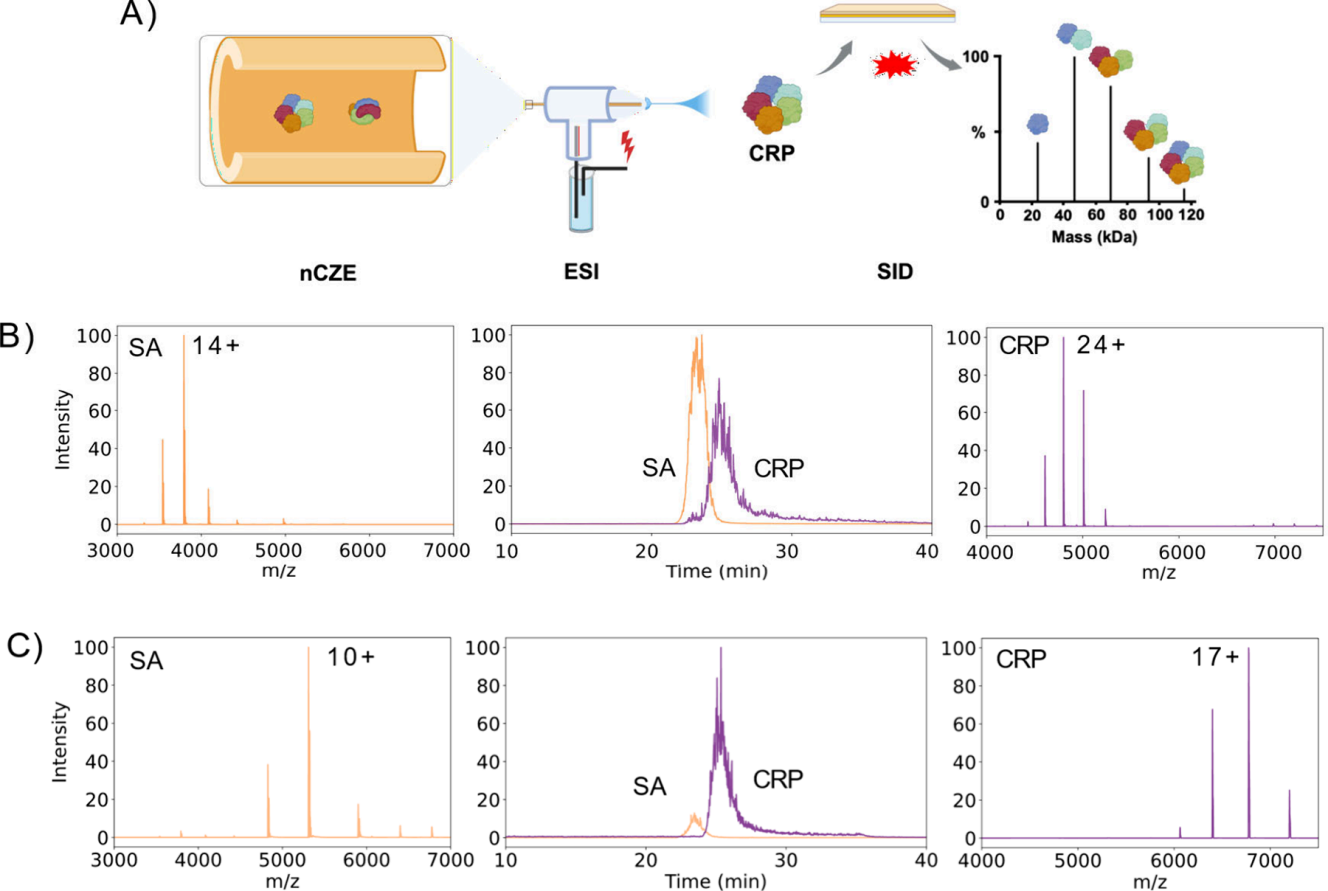
(A) Schematic diagram of nCZE-MS/MS by SID. The figure was created using the BioRender. (B) Electropherogram and MS under normal charge conditions. (C) Electropherogram and MS under charge-reducing conditions.

Figure 1A shows the schematic diagram of our experimental setup. We optimized the peak widths of the migration times so that two protein complexes could be well-separated while still allowing enough ions to be collected for fragmentation. For the normal charge conditions, the charge states of SA center around 14+, and the charge states of CRP center around 24+, Figure 1B. For charge-reducing conditions, the charge states of SA center around 11+ while the charge states of CRP center around 17+, Figure 1C. The charge state distributions in nCZE-MS and direct infusion nMS generally agree with some slight differences, Figure S3. For example, for the normal charge condition, the most abundant charge state of CRP is 23+ for direct infusion nMS and 24+ for nCZE-MS. We further analyzed the charge state distributions of CRP under normal and charge-reducing conditions at different periods of the electrophoretic peak, revealing consistent charge state distributions for the nCZE-MS data, Figure S4.

While the mechanisms of charge reduction are not fully understood, a general mechanism of charge reduction by adding electrolytes in ESI, named the charge carrier-field emission model (CC-FEM), was recently shown. According to this model, charge-reducing reagents undergo ion evaporation during ESI, and the overall charges of the ESI-generated droplets decrease; with further desolvation, the net charge of the protein complex decreases.^41^ Our nCZE-MS results support the idea that charge reduction happens in the ESI process rather than in the solution phase. The charge-reducing condition yielded slightly longer migration times of the two protein complexes compared to the normal condition, Figures 1B,C and S5. The migration time differences between normal and charge-reducing conditions are about 0.3 min (SA) and 0.5 min (CRP), Figure S5, which is approximately 3 and 5 times larger than the standard deviation of migration time of the protein complexes across triplicate nCZE-MS runs. The data indicate that the charge-reducing condition may slightly reduce the charge-to-size ratios (i.e., electrophoretic mobility) of SA and CRP during nCZE. However, according to the migration time of SA and CRP, the electrophoretic mobility reduction in the charge-reducing condition is only approximately 2%. The most abundant gas-phase charge states of SA and CRP were reduced by nearly 30% in the charge-reducing condition. The results suggest that the charge reduction induced by TEAA happens during the ESI process rather than in the solution phase.

During the nCZE-MS process, SA undergoes a signal decrease of 1 order of magnitude after switching BGE to the TEAA-containing solution, but CRP does not undergo a signal decrease after changing the BGE. This phenomenon was also observed with direct infusion (Figure S2), an observation that we cannot currently explain but that might be related to the surface activity of CRP vs SA in the droplet.

**Figure 2:**
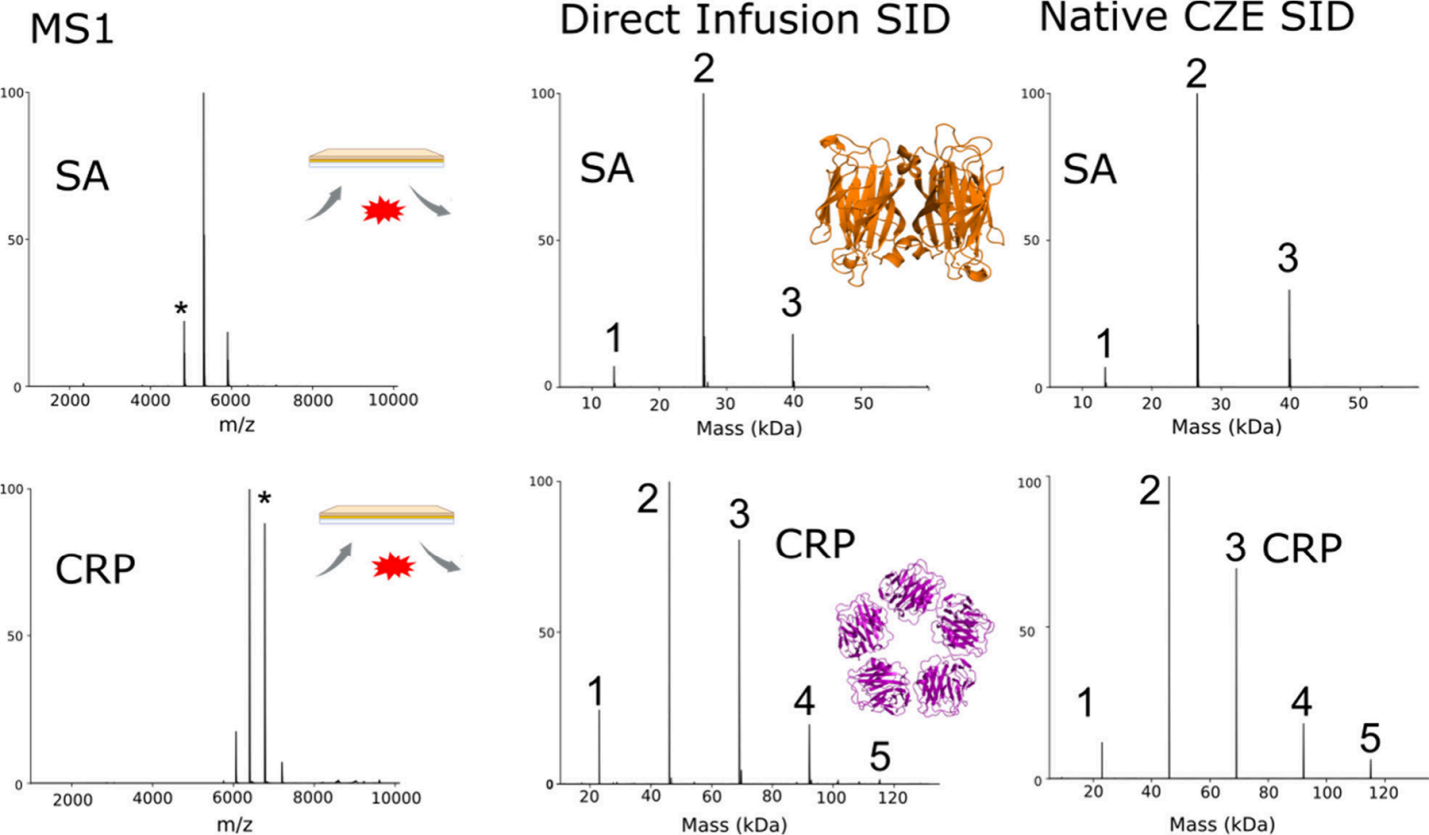
SID of standard protein complexes after nCZE-MS under charge-reducing conditions shows native-like fragmentation results similar to those by direct infusion nMS. The published three-dimensional quaternary structures are shown as insets in the middle panel (PDB code 1SWB for SA and 1GNH for CRP).

The main goals of this work are to generate SID fragmentation patterns for protein complexes as they are separated by CZE and to assess how native-like the CZE-produced precursors are. SID fragments protein complexes along their weakest interfaces.^18^ Changes in the proteins’ structures can greatly affect the resultant fragmentation pattern. As a result, we use SID as a diagnostic tool to evaluate the topology of CZE-separated protein complexes.^21^ A comparison of SID fragmentation patterns under charge-reducing conditions for both direct infusion and for nCZE-SID is shown in Figure 2.

Under both normal charge (Figure S6) and charge-reducing (Figure 2) conditions, sampling the entire peak of nCZE-MS and the direct infusion nMS peak produced similar SID patterns, and the observed fragment populations agree more with native-like fragmentation than with collapsed complexes based on what has been previously reported^22^ (Figure S7). Especially with the BGE containing TEAA, the SID patterns are consistent with the published PDB structures. For SA, SID shows a dominant dimer population and a lower abundance of monomer and trimer, matching its dimer-of-dimers topology, where the interface between two dimers is cleaved at low surface collision energies.^26^ For CRP, we observe a distribution of all oligomeric states from monomer to pentamer, with dimer and trimer being the dominant species. The populations generated by nCZE-MS-SID and direct infusion-SID generate fragmentation patterns indicative of native-like fragmentation.^39,40^ Both SID results for CRP match what we expect for a ring-shaped homo-oligomeric protein, where all oligomers (monomer plus tetramer, dimer plus trimer) are generated upon surface collision due to the equal interfaces between each protomer.^40^ The general agreement of the SID fragmentation patterns with the published structures and previously reported SID fragmentation patterns suggest that the overall quaternary structure of these protein complexes is minimally disrupted by travel through the capillary under a relatively strong electric field. The data indicate that nCZE-MS can maintain the native-like structural topology of the two model protein complexes under our experimental conditions.

To quantitatively evaluate the similarity between the SID fragments generated by nCZE-MS and direct infusion, we used a similarity index (SI), a typical statistical comparison between spectra.^42,43^ When two spectra are identical or closely similar, the calculated SI is close to 1. Table S1 summarizes the abundance generated by UniDec and the corresponding SI. For both protein complexes, under both conditions, the SIs are no less than 0.95 (0.95 for normal charge SA, 0.98 for charge reduced SA, 1.0 for normal charge CRP, 0.99 for charge reduced CRP), suggesting a high similarity between the SID fragments generated by nCZE-MS and direct infusion. This analysis further enhances our conclusion that nCZE-MS does not disrupt the native-like topology.

Visible slight differences were observed in the SID fragmentation patterns of the protein complexes between direct infusion and nCZE, Figure 2. For example, we observed a stronger signal of CRP pentamer precursor in the nCZE SID data than in the direct infusion SID data and slightly more monomer and trimer in the direct infusion SID than nCZE SID. We attribute those slight differences to the fact that different structural conformations or different proton distributions^44^ of the protein complexes could be separated by nCZE. As shown in Figure S8 for CRP, we observed a trend of decreasing trimer and pentamer and increasing dimer across its electrophoretic peak. The data suggest that even though a single charge state (17+) was isolated for SID, nCZE-MS-SID could resolve conformational or charge site differences of protein complexes. With the same similarity analysis stated above, different conformations/proton locations are similar (SI = 0.97) but with a lower similarity level than the difference between sampling the entire peak for nCZE and direct infusion (SI = 0.99), Figure S9. We will perform more systematic studies in this direction in the future.

## Conclusions

In our work, we show the first example of integrating nCZE-MS and SID for protein complexes. Because SID fragmentation patterns generated by nCZE-MS and direct infusion reflect protein complexes whose native quaternary structure has been maintained, we conclude that the two protein complexes (SA and CRP) separated by CZE retain their native-like topologies under our experimental conditions. We additionally show that TEAA does not substantially affect the size-to-charge ratio of the protein complexes in the solution because charge reducing conditions do not drastically affect the migration time of either protein complex in our mixture. We therefore conclude that the charge reduction takes place mainly during the ESI process. It remains possible that some protein complexes with low stability may have their quaternary structures perturbed substantially by CZE separation, which needs to be studied further with a larger number of standard protein complexes. Based on this pilot study, we predict that nCZE coupled to native MS and MS/MS with SID could become a powerful analytical tool for interactomics and structure omics.

## Supporting Information Available

Experimental setup of quad isolation; signal comparison with different electrolyte conditions under different ionic strengths by direct infusion; comparison between MS1 charge state envelope for both nCZE-MS and direct infusion under different electrolyte conditions; comparison between MS1 charge state envelope for direct infusion and nCZE-MS under different electrolyte conditions and different time window within an electrophoretic peak of CRP; extracted ion electropherogram for both protein complexes under normal charge and charge-reduced conditions; deconvolved SID MS/MS for two protein complexes under normal charge condition; comparison between nCZE-MS-SID, direct infusion SID and published collapsed conformation for CRP; deconvolved SID MS/MS for CRP under charge-reduced condition for different sections of the electrophoretic peak; similarity comparison of two deconvolved SID MS/MS under the same electropherogram peak; and the abundance of each fragment generated by SID and similarity index calculation (PDF)
