# Supplementary material for: Does Native Capillary Zone Electrophoresis-Mass Spectrometry Maintain the Structural Topology of Protein Complexes?
Source: Anal Chem. 2025 Apr 1;97(14):7616–21. doi: 10.1021/acs.analchem.4c06949 (PMC12004354; doi:10.1021/acs.analchem.4c06949)
Supplement: Supplementary file 1 — ac4c06949_si_001.pdf [file ac4c06949_si_001.pdf]

Supporting Information

Does native capillary zone electrophoresis-mass spectrometry maintain the structural topology of protein complexes?

William J. Moeller[1,2 #], Zihao Qi[1,2 #], Qianjie Wang[3], Qianyi Wang[3], Vicki H. Wysocki[1,2,*], Liangliang Sun[3,*]

[1]Department of Chemistry and Biochemistry, The Ohio State University, Columbus, Ohio, 43210, United States.

[2]Native MS Guided Structural Biology Center, The Ohio State University, Columbus, Ohio, 43210, United States.

[3]Department of Chemistry, Michigan State University, 578 S Shaw Lane, East Lansing, Michigan, 48824, United States.

# These two authors contributed equally to this work.

* Corresponding authors.

Liangliang Sun, email: lsun@chemistry.msu.edu

Vicki H Wysocki, email: vwysocki3@gatech.edu

Table of Contents

Figure S1........S3
Figure S2........S4
Figure S3........S5
Figure S4........S6
Figure S5........S7
Figure S6........S8
Figure S7........S9
Figure S8........S10
Figure S9........S11
Table S1........S12

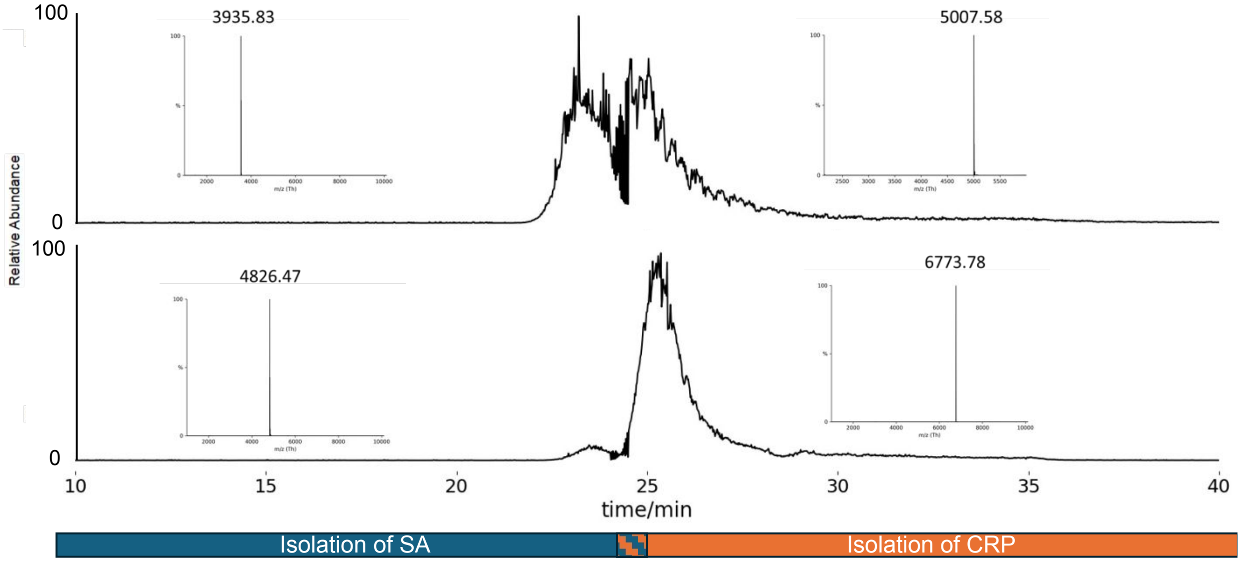

Figure S1. The total ion chromatogram (TIC) with a schematic representation of isolation under both normal charge conditions (top) and charge-reducing conditions (bottom). Quad isolation windows were set based on *m/z* values determined in previous experiments. Quad isolation alternates between species of interest every other scan during the periods when migration times overlap.

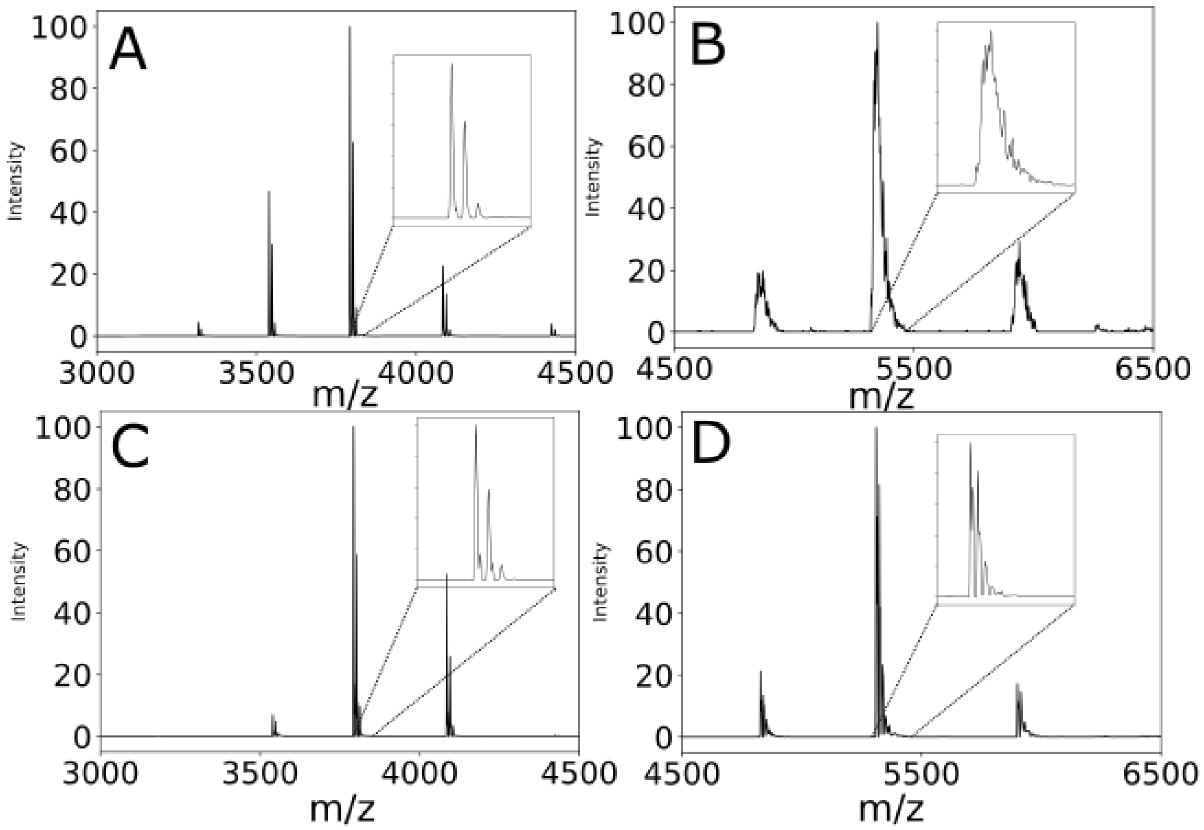

Figure S2. Comparison of ionic strengths for nMS-based experiments under normal charge and charge-reducing conditions for SA. A+C) SA sprayed under normal charge conditions in 20 mM AmAc (A) and 50 mM AmAc (C). B+D) SA under charge-reducing conditions in 16 mM AmAc with 4 mM TEAA (B) and 40 mM AmAc with 10 mM TEAA (D). The signal read back from Xcalibur are, respectively, (A). 3.24e5, (B). 8.10e3, (C). 5.16e5, and (D). 6.12e4.

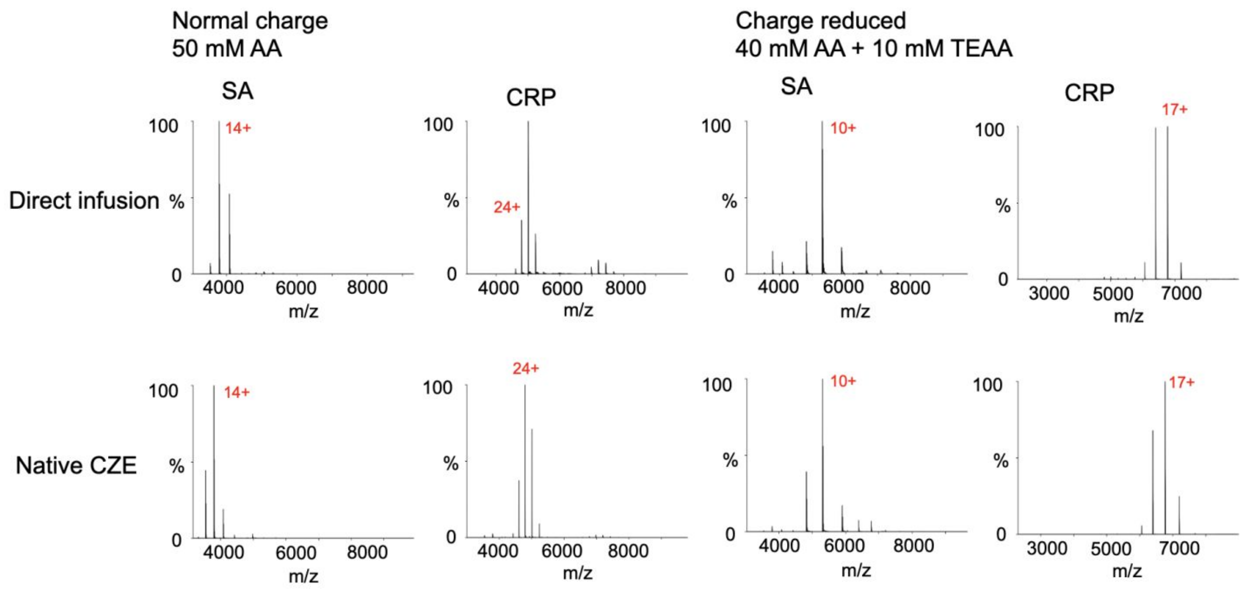

Figure S3. Comparison of MS1 for direct infusion nMS (top row) and native CZE MS (bottom row) for both SA and CRP under both normal charge (left) and charge-reducing (right) conditions.

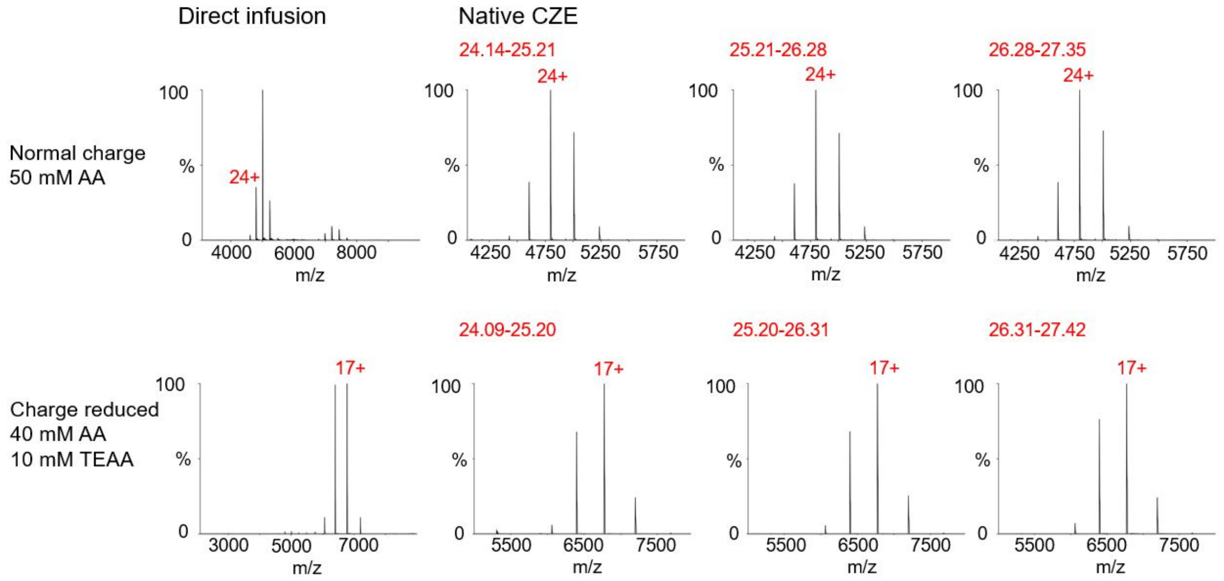

Figure S4. Comparison of MS1 for direct infusion nMS and nCZE MS for CRP by averaging different periods of the CRP electrophoretic peaks for both normal charge and charge reduced conditions.

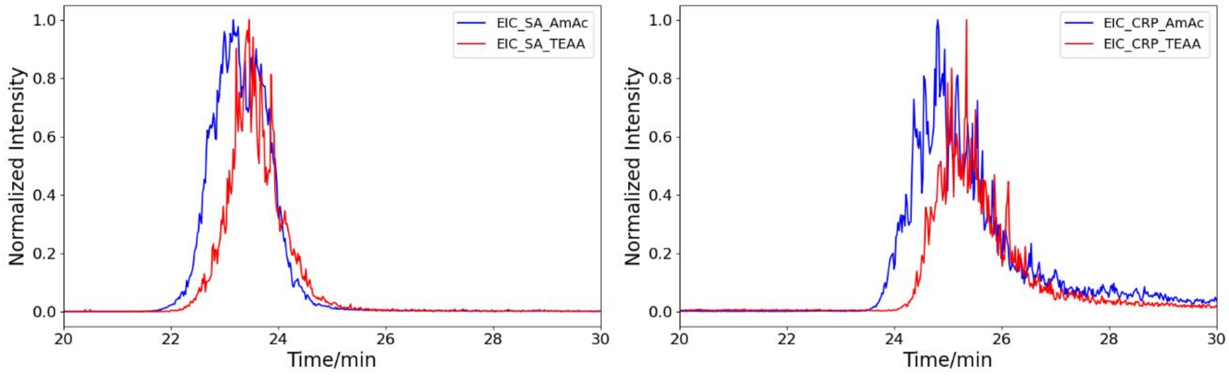

Figure S5. The overlayed EIC of a single charge state (with additives) for both SA (left) and CRP (right) under normal charge (blue) and change-reducing (red) conditions. The intensity is normalized to the highest values. The specific apex values after smoothing of EIC are shown below: SA in AmAc: 23.2 min; SA in AmAc + TEAA: 23.5 min; CRP in AmAc: 24.8 min, CRP in AmAc + TEAA: 25.3 min.

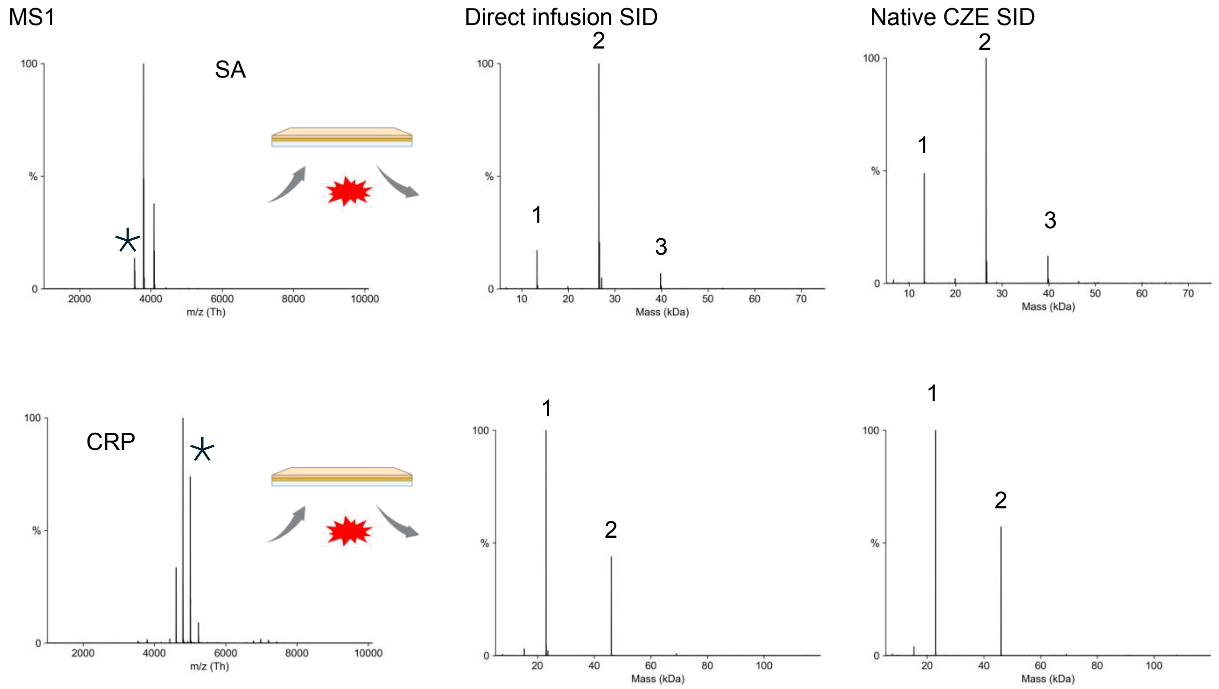

Figure S6. SID of standard protein complexes after nCZE-MS under the normal charge condition shows fragmentation results that agree well with those by direct infusion nMS.

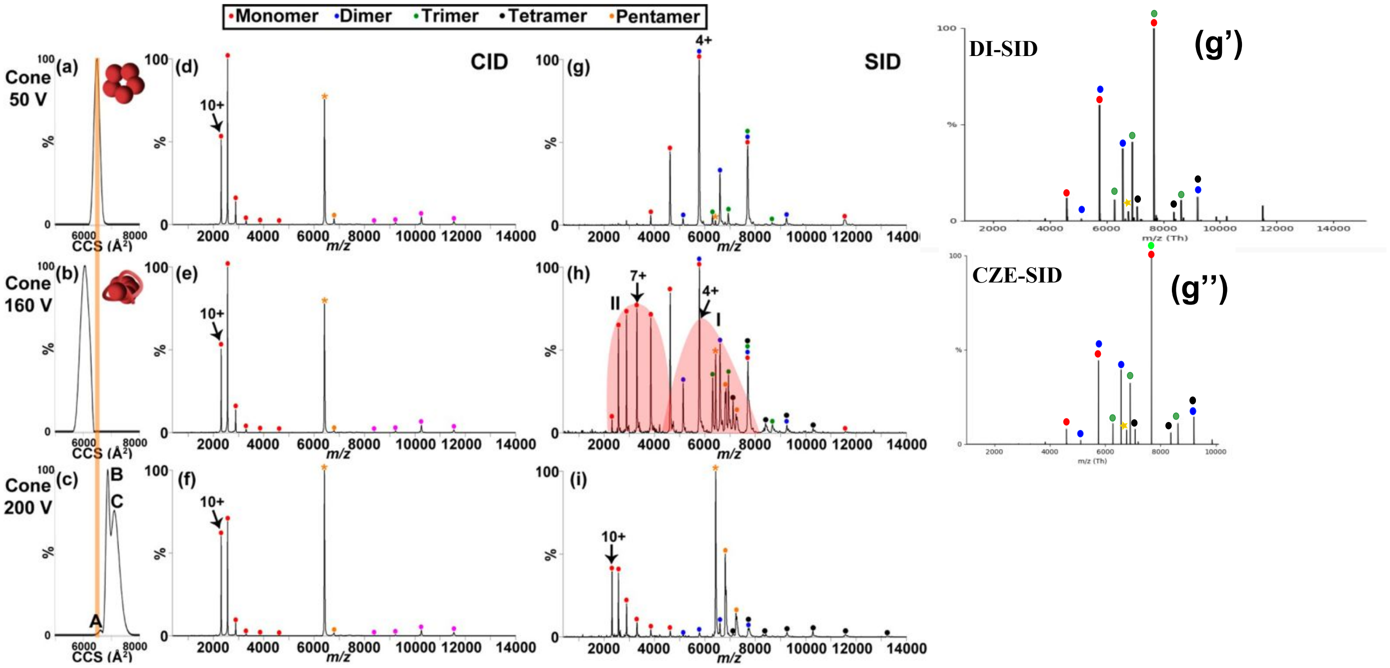

Figure S7. **S7-g** shows the SID of CRP with the ring in its native state, while **h-i** shows a fragmentation pattern generated by the high source activation of protein to purposefully disrupt the native structure. **g'** and **g''** shows the SID fragmentation pattern generated by direct infusion and nCZE-MS, respectively. Figures **g'** and **g''** show far greater agreement with **g** than **h-I,** suggesting that nCZE-MS largely maintains the native structure. Figure modified from Quintyn, R. S.; Zhou, Z.; Yan, J.; Wysocki, V. H., Surface-induced dissociation mass spectra distinguish different structural forms of gas-phase multimeric protein complexes, *Analytical Chemistry,* **2015**, 87, 11879-11886

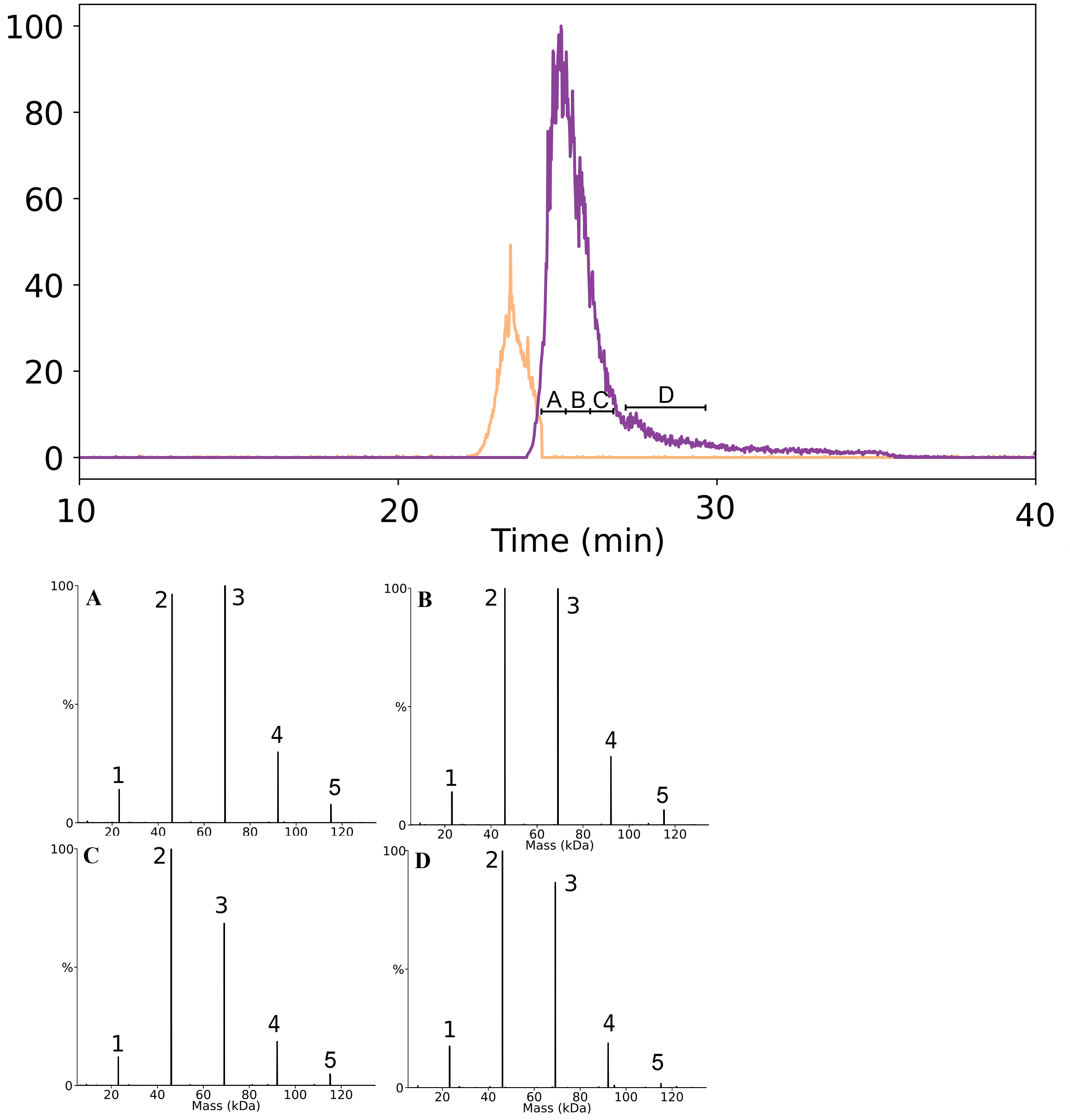

Figure S8. TIC of charge reduced standard protein complexes via nCZE-MS-SID and the deconvoluted mass spectra of CRP data. Deconvolved mass spectra of SID data of CRP at different periods of the electrophoretic peak are shown. Letter A-D correspond to the respective regions highlighted in the electropherogram.

	monomer	dimer	trimer	tetramer	pentamer
front	13.61	94.5	100	29.22	3.86
back	11.86	100	69.3	18.33	3.22

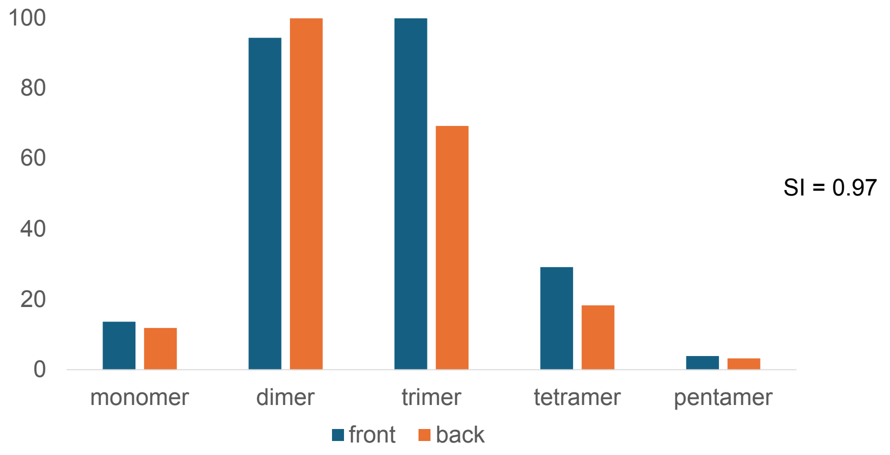

Figure S9. Deconvolved mass spectra of SID data of CRP at two representative periods (front and back correspond with regions A and C as shown in Figure S7) of the electrophoretic peak. SI refers to the similarity index.

Table S1. Summary of a table comparing the similarity of subcomplexes generated by SID under direct infusion (DI) and native CZE (CZE) in both normal charge and charge-reduced conditions for both CRP and SA.

normal charge							
	CRP	monomer	dimer	trimer	tetramer	pentamer	Similarity Index
DI		100.00	41.47	0.00	0.00	0.00	1.00
CZE		100.00	47.12	0.00	0.00	0.00	
DI	SA	20.52	100.00	5.85	0.00	N/A	0.95
CZE		58.39	100.00	11.18	0.00	N/A	
charge reduced							
	CRP	monomer	dimer	trimer	tetramer	pentamer	Similarity Index
DI		24.40	100.00	90.07	20.76	1.04	0.99
CZE		12.71	100.00	82.62	25.26	3.16	
DI	SA	6.51	100.00	9.37	0.00	N/A	0.98
CZE		9.73	100.00	27.14	0.00	N/A	
